# 

**Published:** 1887-01

**Authors:** 


### INDEX TO VOLUME XLI.

A Method of Preparing Dental Tissues for Microscopical Examinations.................. 19
Apparent Recovery from Phthisis.................................. 46
Anæsthesia........................... 61
Alveolar Abscess........................ 73
An Interesting Meeting........... 105
A Change of Position............... 107
A Special Request.................. 108
A New Motor for the Dental Profession.............................. 160
A Ten Minute's Sermon on Nutrition.......................... 169
An Act to Regulate the Practice of Dentistry in Alabama.... 199
A New Method for the Restoration of Respiration Lost under Chloroform............ 207
A Well-Deserved Honor.......... 212
Antiseptic Collodion............... 308
American Dental Association.... 314
A New Record Diagram........... 315
Action of Saline Purgatives...... 344
American Dental Association Meeting.............................. 364
A New Syringe..... ................. 365
Association of the Boards of Dental Examiners............ 368
Address..................................... 437
Address on Dental and Oral Surgery........................... 369
Address by Dr. Kate. C. Moody 378
Address by Dr. J. T. McMilen... 392
An Apparatus for the Relief of Writer's Cramp................ 400
A Story of Science.................. 403
Antiquity of Mercury...... ...... 405
Acids in Digestion................... 414
A New Method of Producing Local Anæsthesia of the Skin............................... 504
A Move in the Right Direction 565
A Case of Tetanus Cured by Hypodermic Injections of Cocaine.................... ........ 567
An Unusual Opportunity......... 575
Bibliographical...................... 158
Blood Poisoning...................... 457
Bacteriology........................... 463
Brown Bread.......................... 516
British Medical Temperance Association....................... 517
Corydon L. Ford, M.D., D.D.S. 5
Compound Fracture of the Superior Maxillary Bone...... 30
Correspondence......................... 53
Correspondence....................... 144
Correspondence...................... 309
Cohesive Foil........................... 117
Crown and Bridge Work......... 141
Calendula as a Styptic............ 309
College Commencement Exercises.............................. 351
Conditions of Membership in the Ninth International Medical Congress.............. 366
Cocaine as a Local Anaesthetic 395
Cocaine Anaesthesia............... 465
Central University of Kentucky 410
Dentistry not a Specialty of Medicine.......................... 21
Diet in Acute Febrile Diseases... 241
Detroit Dental Society............. 256
Druggists' Orders.................... 398
Dentistry in Germany............. 404
Difference Between Creosote and Carbolic Acid.................. 420
Dr. Geo. Watt ....................... 472
Editorial.................................574, 105
Erythroxylon Coco and its Alkaloid, Cocaine................. 331
Educational ........................... 520
Elementary Life...................... 80

3375-37

### INDEX.

Force of Growing Plants......... 37
Food for Reflection..... ........... 345
Food Adulteration.................. 448
Fatal Hemorrhage from Tooth Extraction ........................ 467
Facial Neuralgia..................... 484
Female Medical Students......... 570
Georgia State Dental Society.... 257
Give the Babies Water............ 399
Historical Sketch of the Mississippi Valley Society of Dental Surgeons............... 131
Hospital Reports..................... 300
Hemoptysis—Error of Diagnosis 399
Hygienic Handwriting........... 402
Hygiene in Early Youth... ..... 405
How to Give Iron..................... 414
Hemorrhagic Diathesis............ 469
Hints on Some Points in Eye Diseases Occurring in Childred....... ........................ 512
Hare-Lip Operation................ 517
Is Dentistry a Specialty in Medicine?............................ 24
Is Life Force Matter?.............. 112
International Dental Congress... 146
International Medical Congress Trans-Atlantic Rates......... 152
Irregularities......................... 161
Irregularities......................... 216
Illinois State Dental Society..... 260
International Medical Congress .................. 316, 406, 417, 363
Important Metallurgical Discoveries of a Physician...... 344
Incubation and Transmission of Epidemic Parotitis............ 395
Increase of Blindness in the United States................. 569
In Memoriam........................ 575
Iron Lemonade....................... 570
Kentucky State Dental Examiners............................. 258
Left-Handedness..................... 44
Lister's Latest Antiseptic... ..... 208
Louisiana State Dental Association............................ 56
Microbes.............................. 36
Mississippi Valley Association of Dental Surgeons............ 182
Minutes of Mississippi Valley Association of Dental Surgeons...... ....................... 192
Michigan State Dental Society.. 239
Mississippi State Dental Association.......................... 255
Missouri State Dental Society... 256
Michigan State Dental Society 272
Mania for Novelties in Medicine 298
Medical Cases in Court........... 307
Medical Practitioners and Dentists................................ 453
Medical Congress—Dental Section................ .............. 521
Method for Deodorizing Idoform.............................. 524
Monograph of the First Permanent Molar..................... 529
National Association of Dental Examiners...................... 368
Napelline in Facial Neuralgia... 254
Northern Ohio Dental Society... 260
Notes on Dental Hygiene........ 284
Ninth International Medical Congress....... 673, 415, 585, 535
New England Dental Society.... 491
New Remedies........................ 179
Operative Dentistry............... 8
On the Value of Hemorrhage in the Treatment of Wounds.. 396
Obituary ........ 54, 56, 472, 410, 629
Ohio State Dent. Soc... 524, 471, 574
Prophylaxis more Important than Remedial Treatment.. 39
Proceedings of Cleveland Dental Society....................... 108
Porceedings of Societies.......... 151
Parotitis—Mumps.....................295
Parotid Tumor ....................... 297
Physiological Action of Nitrous Oxide Gas........................ 302
Papayotin in Fissures of the Tongue.......................... 303
Preparation of Tissues for Examination with the Microscope............................ 336
Peroxide of Hydrogen...... ...... 349
Pure Chromic Acid................. 349
Prominence of the Mectric System.................................... 568
Pyorrhoea Alveolaris with Practical Cases....................... 577
Removal of Necrosed bone by Irrigation with Hydrochloric Acid........................ 453
Radical Cure of Alveolar Abscess by Injection of Gutta Percha Solution .............. 59

INDEX.

Rates to Niagara Falls............ 365
Recent Advances in Preventative Med........................ 388
Registration Blank.................. 415
Right Upper Canine Tooth in the Left Orbit.................. 342
Removal of Right Half of Lower Jaw by Slow Enucleation 564
Rumination in Man................ 255
Read Medical Journals............ 568
Some Disorders of Adolesence... 32
Sir Joseph Lester's Operations.. 38
Surface Filth as a Medium of Disease.......................... 45
Salmagundi... 47, 210, 101, 261, 311, ...... 153, 359, 470, 571, 411, 629
Syphilis—Its Dangers and Results .............................. 57
Surgical Aphorisms.................. 94
Syphilis—Signs, Symptoms and Treatment ........................ 125
Surgical Aphorisms.................. 204
Saliva.................................... 213
Something More about Cocaine 251
Submaxillary Calculus............ 252
State Board of Dental Examiners.................................. 259
Southern Dental Association..... 259
Secondary or New Formation of Teeth.......................... 268
Should Physicians Extract Teeth?............................. 304
Status of Dentistry ................ 329
Salivary Fistula Cured by the Galvano-Cautery.............. 343
Salivery Calculus .. ............. 402
Specialty of Dentistry............. 433
Simple Fracture of the Lower Jaw.................................. 509
Science of Hypnotism.............. 519
Tin and Gold Combined as a Filling Material................ 16
Two Cases of Reunion of Cut off Fingers......................... 36
The Importance of Cleanliness.. 38
The Field of Comparative Dentistry.................................. 85
The Wonderful Drug Clerk...... 97
Tumors of the Mouth.............. 109
The Teeth as Dermal Appendages................................. 119
The Relation of Dentistry and Medicine........................... 175
The Dental Law of Indiana as Amended in 1887.............. 201
Talk on the Materials and Methods Used in Taking Impressions ............................ 229
Treatment of Harelip and Cleft Palate............................. 235
The True Law of Population Based upon Physiology and Psycology......................... 244
The Use and Abuse of the teeth 265
The Irradiation of Motor Impulses............................. 282
Tumor of the Parotid Gland..... 286
The Importance of a Liberal Professional Education in the Practice of Dental and Oral Surgery..................... 317
The Advertisements of Religious Newspapers............. 348
The American Medical Association...... ......................... 367
The Southern Dental Association 368
The Influence of Disease upon Population...................... 399
The Effects of Ether on Living Nerves................ .......... 447
Temporary Teeth.................... 421
Treatment of Alveolar Abscess by the Jennings Method.... 430
The Prevention of Cholera Infantum and Kindred Diseases.............................. 461
The Modern Tendency of Disease................................. 468
The Human Nose is a Wonderful Instrument................. 569
Treatment of Stomatitis........... 575
The Congress.................... ..... 574
The American Medical Association................................. 361
University of Tennessee........... 409
Wisconsin Dental Society......... 261
Women in Dentistry............... 525
Warm Ether as an Anaesthetic 567

### Louisiana State Dental Association.

The annual meeting of this body will be held in Tulane Hall, New Orleans, La., on the 23d, 24th, and 25th of February, 1887.

A cordial invitation is extended to the members of the profession throughout the States to attend.

No efforts will be spared to make our guests welcome and comfortable, and the meeting interesting and profitable. Favorable railroad rates may be had at that time; also an opportunity to witness the *Mardi Gras* festivities which take place the day before the meeting.

We anticipate and hope that a large number will be present.

For further information, address

G. J. Friedrichs,
*Chairman Executive Committee,*
153 Carondelet street, New Orleans, La.

### Obituary.

### CONTENTS.

COMMUNICATIONS.

Corydon L. Ford, L.L.D., M.D., D.D.S. ..... 5

Operative Dentistry ..... 8

Tin and Gold Combined as a Filling Material ..... 16

A Method of Preparing Dental Tissues for Microscopical Examinations ..... 19

SELECTIONS.

Dentistry Not a Specialty of Medicine ..... 21

Is Dentistry a Specialty of Medicine? ..... 24

Compound Fracture of the Superior Maxillary Bone ..... 30

Some Disorders of Adolescence ..... 32

Microbes ..... 36

Two Cases of Reunion of Cut-off Fingers ..... 37

Force of Growing Plants ..... 37

Sir Joseph Lister's Operations ..... 38

The Importance of Cleanliness ..... 38

Prophylaxis more Important than Remedial Treatment ..... 39

Left-Handedness ..... 44

Surface Filth as a Medium of Disease ..... 45

Apparent Recovery from Phthesis ..... 46

SALMAGUNDI ..... 47

CORRESPONDENCE ..... 53

EDITORIAL.

Obituary ..... 54-56
Louisiana State Dental Association ..... 56

### THE DENTAL REGISTER,

**A MONTHLY MAGAZINE DEVOTED TO THE INTERESTS OF THE**

**DENTAL PROFESSION.**

***Terms Reduced to* $2.00 *Per Year. Single Copies,* 25 *Cents.***

**EDITED BY PROF. J. TAFT, D.D.S.**

—AND—

**Published by SAM'L A. CROCKER & CO., Ohio Dental and Surgical Depot.**

The volume commences in January and closes in December.
The REGISTER being issued monthly is always fresh, therefore Indispensable to the practitioner who desires to keep posted up with the new inventions and improvements which are daily developed. Neither expense nor effort will be spared to give value and variety to its contents. Articles will be illustrated with well executed engravings whenever they will add to the interest or assist to explanation.
Its extensive circulation and frequency of issue make it one of the BEST DENTAL ADVERTISING MEDIUMS in the United States.
All subscriptions must begin with January or July.
Local or special notices, five lines or less, will be inserted for $3 each insertion; over five lines, 50 cts. per line.
All advertising bills payable in advance, or at furthest, after the issue of the first number containing the advertisement. All transient advertisements to be paid for in advance.
☞CONTRIBUTIONS SOLICITED.
Exclusively Editorial Communications should be addressed to the Editor.
The latest number of the REGISTER may be ordered with or without other goods at any time, and all letters should be addressed to

**SAM'L A. CROCKER & CO., Ohio Dental and Surgical Depot, Cincinnati, O.**

